# Right Atrial Mass Secondary to Breast Cancer Metastasis

**DOI:** 10.7759/cureus.59673

**Published:** 2024-05-05

**Authors:** Corbin Walters, Kishen Patel, Dev Jaiswal, Derek Srouji, Neil Agrawal

**Affiliations:** 1 Department of Cardiology, Oklahoma State University Medical Center, Tulsa, USA; 2 Department of Cardiology, St. Francis Medical Center, Tulsa, USA

**Keywords:** cancer metastasis, breast cancer, cardio-oncology, tumor, echocardiography

## Abstract

We present the case of a 42-year-old female with a history of human epidermal growth factor 2 (HER2) receptor-positive breast cancer status post bilateral mastectomy with metastasis to the spine and to the brain, who underwent transesophageal echocardiography (TEE) after outpatient transthoracic echocardiography (TTE) was suggestive of right atrial thrombus in transit. TEE revealed an atrial mass with a pedunculated stalk attached to the inferior right atrium near the inferior vena cava with a necrotic center. These findings were suggestive of an endocardial metastatic mass secondary to her primary breast cancer. The pericardium is the most common site of cardiac metastasis; meanwhile, endocardial involvement is infrequent, occurring in less than 5% of all cardiac metastases. Right atrial masses may cause evidence of right heart failure and thromboembolism of the pulmonary arteries. Treatment focuses on targeted chemotherapy, radiation therapy, and interventions as indicated. In this case, following the diagnosis of a right atrial mass, the patient was discharged the same day to begin outpatient chemotherapy.

## Introduction

Cardiac metastasis should be suspected with the development of pericardial effusion or new heart murmur, arrhythmia, or conduction delay in patients with known cancer, particularly melanoma, lung, and breast. An estimated 15% of all patients with metastatic cancer have cardiac involvement with the pericardium acknowledged as the most common site of metastasis due to malignant lymphatic invasion [1]. Endocardial involvement is exceedingly rare, occurring in less than 5% of all cardiac metastases [2], particularly among breast cancer where estimates are unknown, with a limited number of documented case reports [3-5]. Endocardial masses of the right atrium may result in signs of right heart failure, such as chest pain, dyspnea, elevated jugular venous pressure, body swelling, and pulmonary tumor emboli. Detection of cardiac tumors begins with transthoracic echocardiography (TTE), with further imaging modalities also frequently employed, including transesophageal echocardiography (TEE), cardiac magnetic resonance imaging (cMRI), and positron emission tomography. TTE may provide key diagnostic information, such as structural appearance, functional interactions including hemodynamics, and possible targets of intervention.

## Case presentation

A 42-year-old female with a past medical history of breast cancer status post bilateral mastectomy with metastasis to the spine and to the brain presented to the emergency department after outpatient TTE was concerning for right atrial thrombus in transit and need for further workup (Video 1). 

**Video 1:** Right atrial mass in transit

**Figure 1:**
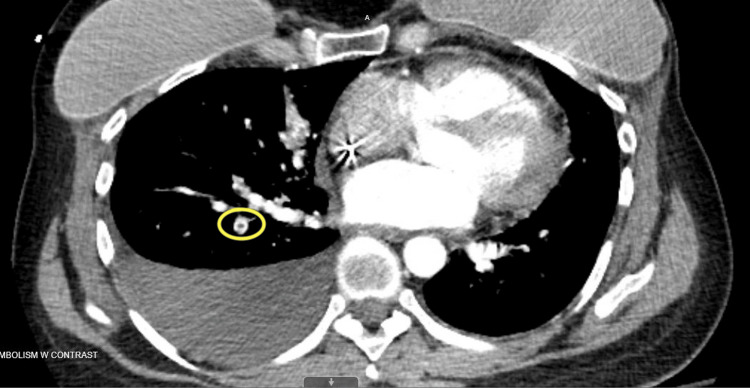
CT pulmonary embolism protocol indicating right lower lobe segmental branch pulmonary artery embolism (indicated by the yellow circle)

Her previously documented immunohistochemistry revealed estrogen receptor (ER)-negative, progesterone receptor (PR)-negative, and human epidermal growth factor 2 (HER2) receptor-positive breast cancer. The patient was asymptomatic without chest pain, shortness of breath, or hypoxia. A CT scan of the pulmonary arteries revealed a segmental right lower lobe branch pulmonary embolism (Figure 1).

**Figure 2:**
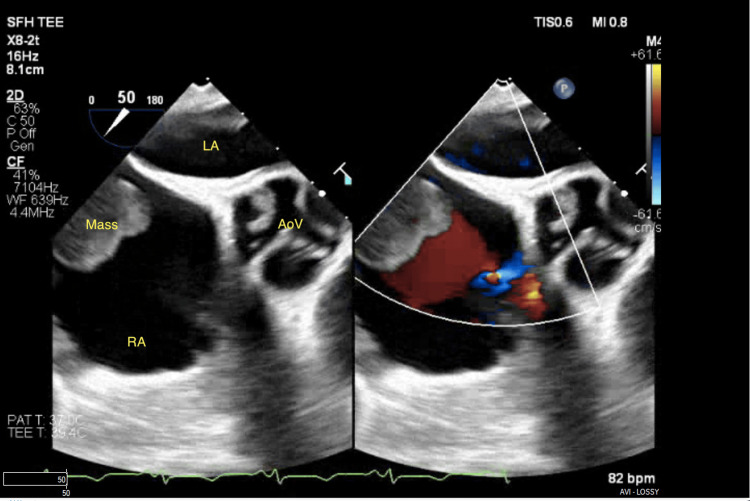
Parasternal short-axis view of the right atrial mass LA: left atrium; RA: right atrium; AoV: aortic valve

**Figure 3:**
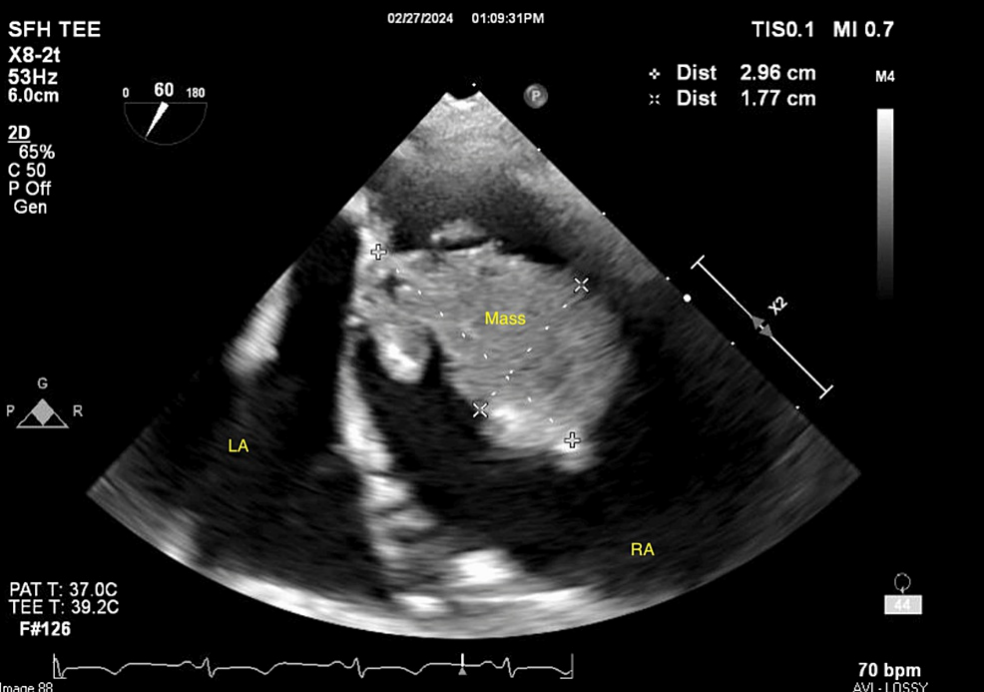
Right atrial mass seen in the bicaval view LA: left atrium; RA: right atrium

**Figure 4:**
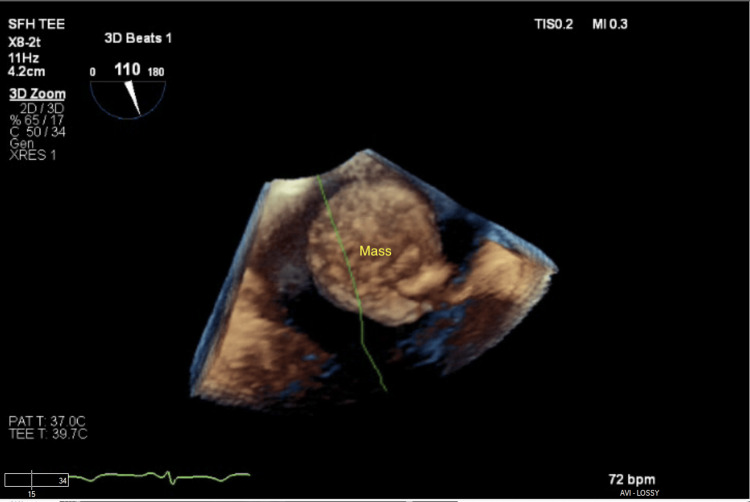
Right atrial mass in three-dimensional view

Given the concern for thromboembolic disease with an adherent clot attached to her recently placed chemotherapy port, TEE followed by clot retrieval was pursued. During TEE, the previously visualized clot was not found to have any interaction with the port; rather it exhibited a pedunculated stalk attached to the inferior right atrium near the inferior vena cava with a necrotic center (Figures 2-4, Video 2).

**Video 2:** Right atrial mass parasternal short-axis view with color Doppler

**Figure 5:**
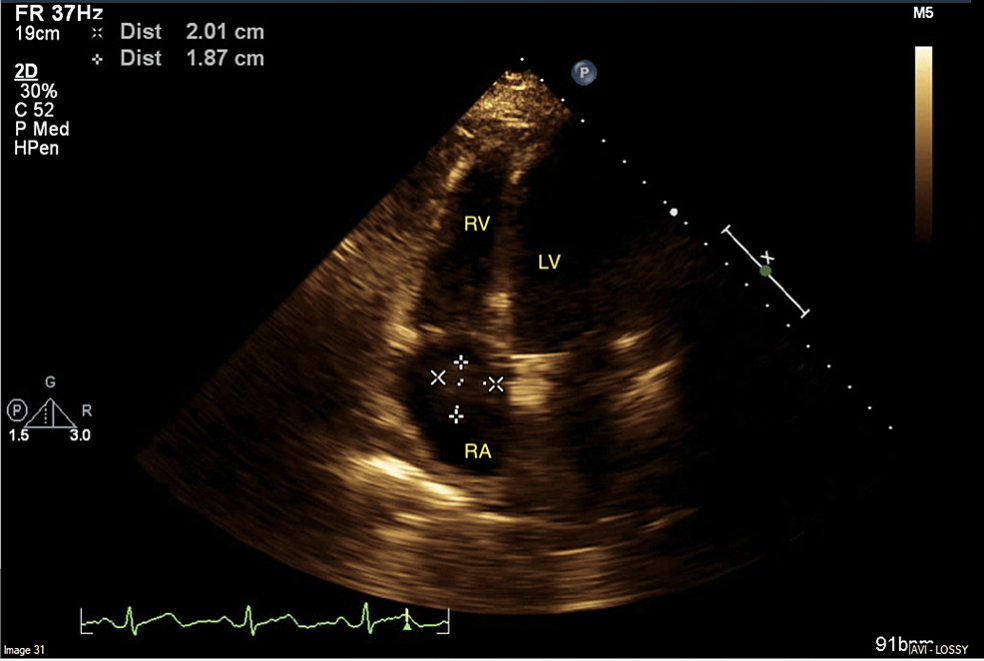
Right atrial mass seen in the apical 4 chamber view RA: right atrium; RV: right ventricle; LV: left ventricle

Given the image findings, the planned retrieval was deferred. After a multidisciplinary discussion, the patient was discharged home on apixaban for the treatment of the pulmonary artery embolism and to begin outpatient chemotherapy the following day. A cardiac MRI has been ordered for the patient to be performed outpatient, but she has been unable to complete this due to multiple hospitalizations, including admission for acute encephalopathy believed to be due to an adverse effect of dexamethasone that is being given with her chemotherapy and a recent admission for severe diarrhea attributed to pertuzumab leading to hematochezia secondary to anticoagulation with apixiban. TTE obtained approximately two months after initiation of chemotherapy shows an interval decrease in the size of the right atrial mass (Figure 5).

## Discussion

The most common primary cardiac tumor is a myxoma, a benign mass, with 20% of tumors arising from the right atrium [6]. Angiosarcomas are the most common primary malignant tumor of the right atrium, presenting with inter-atrial invasion and extension into the pericardium. Cardiac metastasis occurs more frequently than primary tumors; however, endocardial involvement of the right atrium is particularly rare, but it may be seen with aggressive lymphoma and hepatocellular carcinoma. Estimated rates of right atrial involvement following metastatic breast cancer diagnosis remain unknown, with a limited number of documented case reports [3-5]. It is proposed that metastasis occurs via a hematogenous spread using the azygous venous system, which provides transvenous drainage of the breasts, particularly the right breast, to the superior vena cava and cardiac chambers [7]. Echocardiography allows for the inspection of tumor size, site and modality of attachment, shape, surface tissue characteristics, and hemodynamic impact [8]. In fact, 2D TTE tumor measurements have been shown to correlate well with anatomic measurements at the time of surgery [9]. Oftentimes, tumor qualities such as echogenicity, attachment site, stalk size, and mobility can be visualized using 2D TTE along with other enhancement techniques, such as color Doppler imaging, which aids in the differentiation of tumors with low echogenicity, agitated saline, and contrast agents. Further image enhancement and utility can be improved with TEE.

In this case, 2D TTE provided more questions than answers, so TEE was sought for clarification. Intrinsically, the patient is at high risk for hypercoagulability due to her malignancy, and her chemotherapy port serves not only as an attachment for intracardiac thrombi but also as a potential source of infective endocarditis. TEE revealed no evidence of infection or thrombus, but rather a necrotic intracardiac mass attached to the right atrium with a pedunculated stalk, suggestive of breast cancer metastasis.

## Conclusions

The pericardium is the most common cardiac site of metastatic cancer spread with endocardial involvement occurring in less than 5% of all cardiac metastases. The initial imaging modality of choice is 2D echocardiography allowing for the examination of key tumor characteristics, such as size, site and modality of attachment, shape, tissue qualities, and hemodynamic implications. Breast cancer metastasis leading to endocardial infiltration, in particular, is an exceedingly uncommon phenomenon, not regularly compiled statistically. Treatment is focused on targeted therapies for the primary tumor, including chemotherapy, radiation, and surgery when applicable. In the case of metastasis, palliation with management of symptoms is the prevailing consensus. Rarely, surgery may be of consideration in the event of a solitary intracardiac tumor with hemodynamic interference and presumed good overall prognosis, but operative mortality is often high. Thus, shared decision-making is of vital importance. 

In conclusion, this case sought to contribute to the limited literature for identifying metastatic intracardiac lesions echocardiographically.
